# Persistence and baseline determinants of seropositivity and reinfection rates in health care workers up to 12.5 months after COVID-19

**DOI:** 10.1186/s12916-021-02032-2

**Published:** 2021-06-28

**Authors:** Carlota Dobaño, Anna Ramírez-Morros, Selena Alonso, Josep Vidal-Alaball, Gemma Ruiz-Olalla, Marta Vidal, Rocío Rubio, Emma Cascant, Daniel Parras, Natalia Rodrigo Melero, Pau Serra, Carlo Carolis, Pere Santamaria, Anna Forcada, Jacobo Mendioroz, Ruth Aguilar, Gemma Moncunill, Anna Ruiz-Comellas

**Affiliations:** 1 ISGlobal, Hospital Clínic, Universitat de Barcelona, Carrer Roselló 153 (CEK building), E-08036 Barcelona, Catalonia Spain; 2 Unitat de Suport a la Recerca de la Catalunya Central, Fundació Institut Universitari per a la recerca a l’Atenció Primària de Salut Jordi Gol i Gurina, Sant Fruitós de Bages, Spain; 3 Health Promotion in Rural Areas Research Group, Gerència Territorial de la Catalunya Central, Institut Català de la Salut, Sant Fruitós de Bages, Spain; 4 Institut d’Investigacions Biomèdiques August Pi i Sunyer (IDIBAPs), Barcelona, Spain; 5 Biomolecular Screening and Protein Technologies Unit, Centre for Genomic Regulation (CRG), The Barcelona Institute of Science and Technology, Barcelona, Spain; 6 Julia McFarlane Diabetes Research Centre (JMDRC) and Department of Microbiology, Immunology and Infectious Diseases, Snyder Institute for Chronic Diseases, Cumming School of Medicine, University of Calgary, Calgary, Alberta T2N 4N1 Canada; 7 Gerència Territorial de la Catalunya Central, Institut Català de la Salut, Sant Fruitós de Bages, Spain; 8 COVID-19 Response Unit, Department of Health, Generalitat de Catalunya, Barcelona, Spain; 9 Centre d’Atenció Primària (CAP) Sant Joan de Vilatorrada, Gerència Territorial de la Catalunya Central, Institut Català de la Salut, Sant Fruitós de Bages, Spain

**Keywords:** SARS-CoV-2, COVID-19, Antibodies, Spike antigen, Duration, Kinetics, Reinfection, Health care workers, Cohort, Baseline determinants

## Abstract

We assessed the duration and baseline determinants of antibody responses to SARS-CoV-2 spike antigens and the occurrence of reinfections in a prospective cohort of 173 Spanish primary health care worker patients followed initially for 9 months and subsequently up to 12.5 months after COVID-19 symptoms onset. Seropositivity to SARS-CoV-2 spike and receptor-binding domain antigens up to 149–270 days was 92.49% (90.17% IgG, 76.3% IgA, 60.69% IgM). In a subset of 64 health care workers who had not yet been vaccinated by April 2021, seropositivity was 96.88% (95.31% IgG, 82.81% IgA) up to 322–379 days post symptoms onset. Four suspected reinfections were detected by passive case detection, two among seronegative individuals (5 and 7 months after the first episode), and one low antibody responder. Antibody levels significantly correlated with fever, hospitalization, anosmia/hypogeusia, allergies, smoking, and occupation. Stable sustainment of IgG responses raises hope for long-lasting COVID-19 vaccine immunity.

**Supplementary Information:**

The online version contains supplementary material available at 10.1186/s12916-021-02032-2.

## Background

A key question to understand the evolution of the COVID-19 pandemic is the duration of immune response generated to SARS-CoV-2. Most patients induce a robust humoral and cellular response [1] but with high heterogeneity and a percentage of non-responders. Diversity in epitope specificity, quality, and functional capacity of antibodies will likely affect the efficacy of the immunity mediated. Antibodies elicited after exposure to SARS-CoV-2 have been associated with protective immunity up to 6 months [2–5], although we do not yet have a correlate of protection, and reinfections occur seemingly at a low frequency. The spike (S) protein on the virus surface is considered the main target of protective antibodies and the component of the leading vaccines [6] already under implementation. Functional neutralizing antibodies highly correlate with IgG levels to the receptor-binding domain (RBD) of S [1], but IgA and IgM also have neutralizing properties [7].

Despite an increasing understanding of the nature of antibody responses, their longevity remains to be defined as the pandemic evolves. The duration of protective antibodies is a critical question as reinfection rates may increase if immunity wanes. Although initial reports indicated a decline in antibodies after 3 months [8], subsequent studies have shown relatively stable antibody levels, mostly IgG, over a period of up to 6 months and beyond [1–5, 9–16]. As massive global immunization campaigns advance, this knowledge will provide insight as to how long COVID-19 vaccine immunity might last and how preexisting SARS-CoV-2 antibodies and other baseline variables could affect vaccine effectiveness.

We aimed to determine the SARS-CoV-2 seropositivity rate up to 12.5 months after COVID-19, identify clinical determinants of antibody levels, and establish the reinfection rates in a prospective cohort of health care workers (HCW) who experienced COVID-19 during the first wave in 2020. We hypothesized that IgG antibodies to the S antigen will be maintained over positivity thresholds for 1 year in a substantial proportion of participants after recovery from symptomatic disease.

## Methods

### Study subjects

Demographic and clinical data were collected to characterize the factors associated with disease presentation, presence of sequelae, long COVID-19, and reinfection, in a cohort of 173 primary HCW in Barcelona, Spain, recruited during the first peak of the pandemic (March–April 2020) (Additional file 1: Table 1). Study physicians and nurses collected baseline clinical characteristics through telephone interviews, and a nurse performed the clinical follow-up questionnaires on the same day of blood collection. Recorded baseline symptoms included fever, shivers, headache, asthenia, myalgia, arthralgia, dyspnea, chest pain, cough, sputum production, hemoptysis, anosmia, hypogeusia, odynophagia, tachycardia, dizziness, and thrombosis. For the multivariable regression analysis, symptoms were grouped into categories: digestive, otolaryngology, neurological, ophthalmology, and skin disorders. Baseline information collected also included history of previous environmental allergies (pollen, mites, and animal hair) and smoking habits.

Five cross-sectional surveys were performed between September to November 2020, and January to April 2021, to obtain the venous blood for assessing maintenance of anti-SARS-CoV-2 seropositivity and analyze baseline factors associated with antibody levels. Reinfection cases were collected by passive case detection and through the clinical follow-up questionnaires during the cross-sectional visits, as participants were not systematically monitored for potential asymptomatic reinfections. Vaccinated HCW were excluded from this analysis.

### Laboratory analyses

Levels of IgM, IgA, and IgG to RBD and S recombinant proteins expressed from plasmids donated by F. Krammer (Mount Sinai, NY) were quantified in plasma by Luminex [17]. Antigen-coupled microspheres were added in multiplex to a 384-well μClear® flat bottom plate (Greiner Bio-One, Frickenhausen, Germany) in 90 μL of Luminex Buffer (1% bovine serum albumin [BSA], 0.05% Tween 20, 0.05% sodium azide in phosphate-buffered saline [PBS]) using an Integra Viaflo semi-automatic device. Positive control pools were added to each assay plate as serially diluted titration curves for QA/QC purposes. Pre-pandemic samples were used as negative controls. Test and control plasma samples were added to a 384-well plate using an Assist Plus Integra device (test sample dilution at 1:500). For IgM, samples were pre-treated with anti-human IgG (Gullsorb) at 1:10 dilution, to avoid IgG interferences. Technical blanks (Luminex Buffer and microspheres without samples) were added to control for non-specific signals. Plates were incubated for 1 h at room temperature in agitation at 900 rpm and protected from light. Then, 384-well plates were washed three times with 200 μL/well of PBS-T (0.05% Tween 20 in PBS), using a BioTek 405 TS. Twenty-five microliters of goat anti-human IgG-phycoerythrin (PE) (GTIG-001, Moss Bio) at 1:400, goat anti-human IgA-PE (GTIA-001, Moss Bio) at 1:200, or goat anti-human IgM-PE (GTIM-001, Moss Bio) at 1:200 in Luminex buffer was added to each well and incubated for 30 min. Plates were washed and microspheres resuspended with 80 μL of Luminex Buffer and acquired on a Flexmap 3D® reader (at least 50 microspheres per analyte per well), and median fluorescence intensity (MFI) was reported for each analyte. The cutoff for seropositivity was calculated with 128 prepandemic samples as 10 to the mean plus 3 standard deviations of log_10_-transformed MFI values.

### Data analysis

Antibody levels were correlated with days since onset of symptoms, and results expressed as Spearman coefficient (rho) and *p* values. Univariable and multivariable stepwise linear regression models were fit to determine the effect of baseline variables on the antibody levels (log_10_) in the full cohort before the start of vaccination (December 2020). Models were selected based on the Akaike information criterion, Bayesian information criterion, and adjusted R^2^ parameters. A transformed beta value (%) of the log-linear model was calculated with the formula: ((10^beta)-1)*100, giving the difference (in percentage) in antibody levels when comparing to the reference group for categorical variables or for a one-unit change for continuous variables, for easier interpretation of the beta value results. All *p* values were considered statistically significant when <0.05. All data collected were managed and analyzed using the R software version 3.6.3.

## Results and discussion

Most clinical cases in this cohort of HCW were mild-moderate COVID-19, with 24 hospitalized, and 64 presenting with sequelae (Additional file 1: Table 1).

**Fig. 1:**
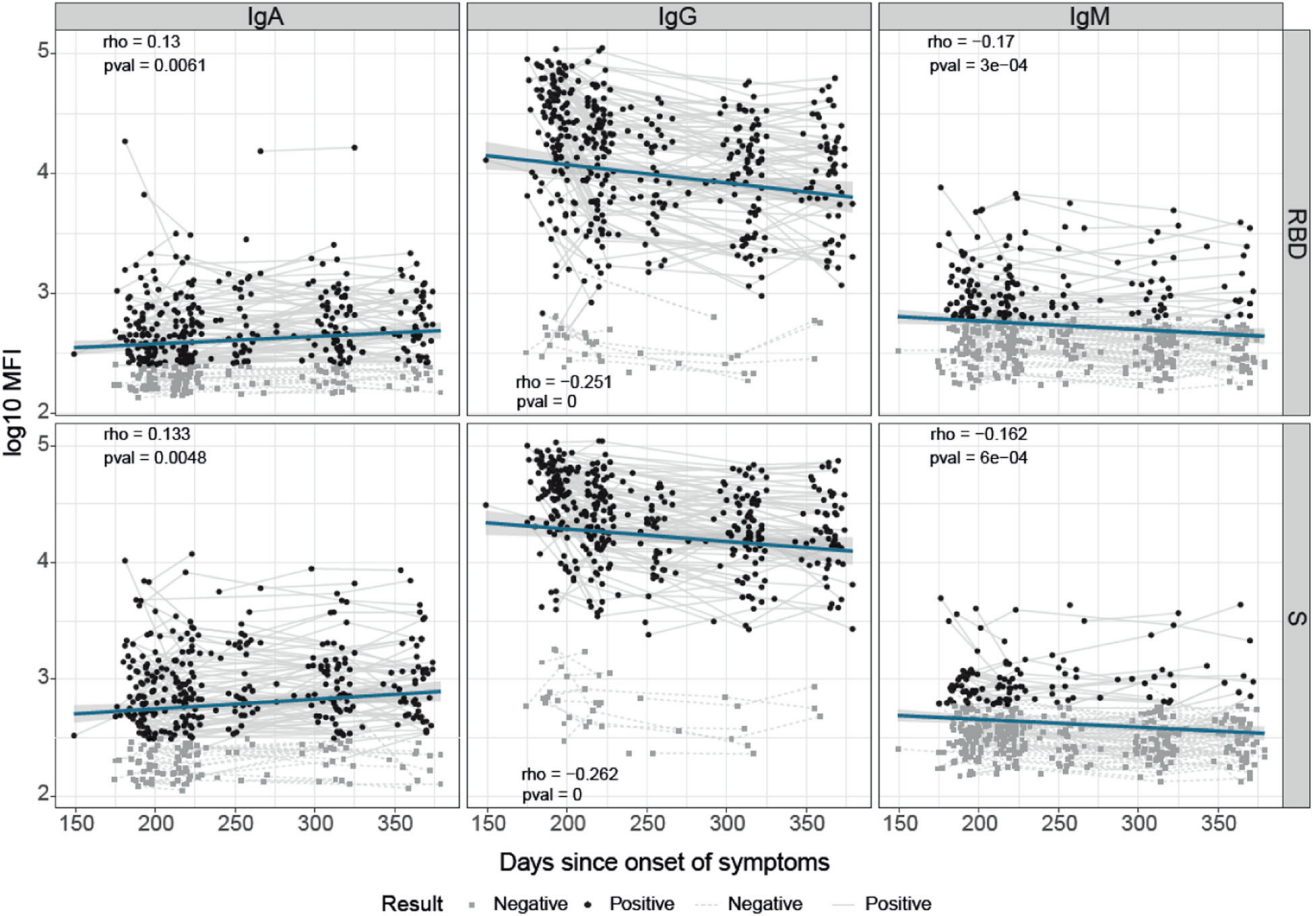
SARS-CoV-2 antibody levels by days since COVID-19 symptoms onset. IgA, IgG, and IgM levels are represented in log_10_ median fluorescence intensity (log_10_ MFI). Black dots represent seropositive individuals, and gray dots represent seronegative individuals. Paired samples are joined by gray lines (continuous lines in seropositive and dashed lines in seronegative). The blue solid line represents the fitted curve calculated using the linear model method. Shaded areas represent 95% confidence intervals

We did not detect a significant decline in antibody levels as a function of time since symptoms onset in the study period at late convalescence, after 5 months (Fig. 1). The percentage of seropositivity 149–270 days after symptoms onset combining RBD and S antigens was 60.69% for IgM, 76.30% for IgA, and 90.17% for IgG, consistent with the expected longer duration of the latter isotype. Unexpectedly, seropositivity was also quite sustained for IgM and IgA, which are considered to be isotypes that have shorter duration. Computing all immunoglobulins, seroprevalence 5–9 months after the initial COVID-19 episode was as high as 92.49%, indicating very stable persistence of responses. The high sensitivity of our Luminex method [17] may contribute to a higher positivity.

Furthermore, 64 of 173 HCW not yet vaccinated were tested in January and April 2021, and the overall percentage of seropositivity up to 322–379 days after onset of symptoms in this subset was still as high as 97% (IgG 95%, IgA 83%, IgM 25%). These 64 HCW had a seropositivity of 98% at 5–9 months after the initial COVID-19 (IgG 95%, IgA 87%, IgM 37%). A high rate of seropositivity up to 9 months post COVID-19 has also been reported in previous longitudinal seroprevalence studies in China [11].

There were four suspected reinfections (Table 1). Before the second positive polymerase chain reaction (PCR) diagnosis, two symptomatic cases were seronegative, one asymptomatic was seropositive with low antibodies, and one had unknown serostatus. We attempted to recover the viral RNA from the first episodes for genome sequencing and demonstration of different strains, but unfortunately, it was not kept stored. This data set provides some indication of the frequency of reinfection in 173 primary infections with three likely reinfections (interval >90 days as per CDC guidelines) and one suspected reinfection (<90 days between primary and reinfection). Therefore, there was a minimal overall rate of symptomatic re-infection of 2/173 (1.16%). This rate contrasts with what we found in another HCW cohort that we followed for a 7-month seroprevalence study in which no reinfections were detected [5, 18]. It could be that primary HCW are more at risk of reinfection than hospital-based HCW, although our study is based on a limited sample size. The study also provides some evidence that a lack of S antibody response is a risk factor for symptomatic reinfection while positive serology leads to asymptomatic reinfection (Table 1). This is relevant due to the strong correlation (rho=0.9) between IgG antibody levels to S and RBD with neutralizing function that are thought to confer protection [5].

**Table 1:** Characteristics of the suspected SARS-CoV-2 reinfection cases

Socio demographics	First COVID-19 episode		Second COVID-19 episode		Serology
	Symptoms^a^	PCR	Symptoms	PCR	
Female, 29-year-old nurse	March 15–May 14	Positive: April 2 Negative: April 22	October 13–December 23	Positive: October 13	Seronegative September/Seroconverted October
Female, 41-year-old physician	March 24–May 25	Positive: March 27 Negative: April 21, May 4	August 2020–Jan 2021	Positive: August 25, September 8 Negative: October 9	Seronegative May & August/Seroconverted September
Female, 58-year-old administrative	March 23–March 25	Positive: March 23 Negative: April 6	May 20–May 22	Positive: May 21, June 4, 11, 18 Negative: June 25	Unknown April/Seropositive November
Female, 44-year-old physician	March 23–April 3	Positive: March 25 Negative: April 4	None	Positive^b^: November 19	Seropositive^c^ September

^a^Date of start and end of the first and last symptoms. All dates are 2020 unless otherwise indicated

^b^PCR was done prior to an unrelated surgical procedure and not as part of any routine COVID-19 screening, the participant had no symptoms

^c^Low-level antibody responses above the seropositivity threshold

Stepwise multivariable regression analyses showed that the baseline factors most consistently and significantly associated with higher levels of antibodies 5–9 months after infection were having been admitted to hospital, presenting fever (*n* = 131), anosmia and/or hypogeusia (*n* = 106), and having had previous allergies (*n* = 24) (Table 2). Specifically, for anti-S IgG, HCW with fever had 2.5 times higher levels, patients with anosmia and/or hypogeusia had 2.6 times higher levels, and those with allergies had 1.9 times higher levels, than patients without those conditions. Baseline factors associated with lower levels of IgA and IgG included being a nurse (*n* = 68) or a physician (*n* = 70) compared to other occupation categories working in primary health care centers including customer and social services staff (*n* = 35), and smoking. For anti-S IgA, physicians had 34.84% and nurses 45.67% lower levels than the other job categories, and smokers had 46.17% less than non-smokers (Table 2). Nurses included eight auxiliary nurses, and physicians included one dentist. Other factors were associated with only certain isotypes. Presenting with sputum and/or hemoptysis (*n* = 13) was associated with higher IgM levels, and shivers (*n* = 86) were associated with higher IgAs. Of note, hospitalized patients had 2.1 times higher IgM levels to RBD than non-hospitalized. Age correlated positively with IgGs, for every incremental increase of age by 1 year, there was a 1.39% increase in IgG levels to RBD (Table 2). This positive association is likely because older people have more serious clinical presentations, being admitted to hospital more often, which is associated with higher antibodies. Higher IgG (and IgA less strongly) levels positively correlated with duration of symptoms (median 24 days, IQR 13–36; S rho=0.229 *P* = 0.002; RBD rho=0.246, *P* = 0.001) and number of symptoms (median 10, IQR 6–12; S rho=0.351 *P* < 0.001; RBD rho=0.364, *P* < 0.001). All other variables, symptoms, or sequelae, were either not statistically significantly associated with antibody levels or weakly associated in univariable models.

**Table 2:** Baseline variables associated with SARS-CoV-2 spike antibody levels 5–9 months after COVID-19 symptoms onset by multivariable stepwise regression models

	Predictors	Spike				Receptor binding domain			
		Beta^a^	95%CI		***P*** value	Beta	95%CI		***P*** value
**IgM**^b^	Hospitalization	0.187	0.025	0.348	0.024	0.324	0.150	0.498	<0.001
	Previous allergies	0.157	0.000	0.314	0.051	ns	ns	ns	ns
	Sputum and/or hemoptysis	0.156	−0.050	0.363	0.137	0.268	0.047	0.489	0.018
	Anosmia/hypogeusia	0.108	−0.003	0.220	0.057	0.091	−0.028	0.210	0.133
	Fever	0.091	−0.038	0.219	0.165	0.112	−0.027	0.250	0.113
	Digestive alterations	ns	ns	ns	ns	−0.089	−0.210	0.033	0.152
**IgA**	Fever	0.250	0.094	0.406	0.002	0.178	0.060	0.296	0.003
	Previous allergies	ns	ns	ns	ns	0.157	0.016	0.298	0.029
	Hospitalization	ns	ns	ns	ns	0.156	0.013	0.299	0.033
	Shivers	0.160	0.024	0.296	0.022	0.087	−0.014	0.188	0.091
	Anosmia/hypogeusia	0.139	0.004	0.273	0.043	ns	ns	ns	ns
	Smoking	−0.269	−0.524	−0.015	0.038	−0.222	−0.411	−0.032	0.022
	Nurses	−0.265	−0.443	−0.086	0.004	−0.223	−0.357	−0.090	0.001
	Physicians	−0.186	−0.360	−0.003	0.046	−0.219	−0.352	−0.087	0.001
**IgG**	Anosmia/hypogeusia	0.413	0.258	0.568	<0.001	0.189	0.077	0.301	0.001
	Fever	0.398	0.218	0.578	<0.001	0.301	0.169	0.432	<0.001
	Previous allergies	0.269	0.053	0.485	0.015	0.137	−0.021	0.295	0.090
	Hospitalization	0.187	−0.024	0.398	0.082	0.236	0.068	0.404	0.006
	Age	0.007	0.000	0.014	0.050	0.006	0.001	0.011	0.023
	Cough	0.124	−0.034	0.283	0.123	ns	ns	ns	ns
	Digestive alterations	ns	ns	ns	ns	0.088	−0.025	0.202	0.126
	Smoking	−0.295	−0.580	−0.009	0.043	ns	ns	ns	ns
	Nurses	ns	ns	ns	ns	−0.187	−0.335	−0.039	0.014
	Physicians	ns	ns	ns	ns	−0.105	−0.253	0.042	0.159

^a^Estimate of the model (beta coefficient), see text for interpretation

^b^log_10_MFI: logarithm 10 median fluorescent intensity (antibody levels)

*CI* confidence interval of the model estimate (beta)

*ns* not significant (not retained in the stepwise forward/backward multivariable model)

Previous acute phase studies showed that COVID-19 severity was associated with higher antibody responses. Here, hospitalization was associated with higher immunoglobulin levels many months after convalescence, suggesting that severity does not affect stability of memory B cell and plasma cells producing antibodies [2–4, 19]. Common symptoms like fever and highly specific symptoms like alteration in smell and taste were also associated with higher antibodies. Interestingly, having previous allergies also positively correlated with higher antibody levels, which to our knowledge has not been reported. This could be related to disease exacerbation and increased risk of respiratory infections associated with some allergies [20] although this relationship remains unclear. Lower antibody levels found in nurses and physicians than other HCW could indicate lower exposure due to personal protective equipment use and higher awareness of risks [18]. Smoking had previously been associated with lower antibody responses [21, 22], and we show that this effect persists after several months primarily affecting IgA, the main mucosal antibody.

## Conclusion

In conclusion, despite the large heterogeneity in antibody levels induced by SARS-CoV-2 infection, most HCW patients remained seropositive for anti-S antibodies up to 12.5 months after COVID-19. The findings that after PCR reversion, 2 out of 13 seronegative individuals had another symptomatic episode, and that one low responder had a second (asymptomatic) infection, are consistent with a protective role of antibodies [23]. Considering that antibody levels achieved by COVID-19 immunization are usually higher than those elicited following natural infection, based on this study, it could be speculated that immune memory induced by the first-generation vaccines could also be long-lasting; therefore, reducing the probability that periodic boosters might be required to sustain protective immunity, at least within the first year. Furthermore, data indicates that naïve people should be prioritized for vaccination over those who had suffered COVID-19, since the latter maintain antibodies for at least a year.

## Additional file 1

**Additional file 1: Table S1.** Baseline characteristics cohort.

## Data Availability

Data and materials are available from the corresponding author upon request.

## Abbreviations

S: Spike
RBD: Receptor-binding domain
HCW: Health care workers
BSA: Bovine serum albumin
PBS: Phosphate-buffered saline
MFI: Median fluorescence intensity
PCR: Polymerase chain reaction
