# Supplementary material for: Persistence and baseline determinants of seropositivity and reinfection rates in health care workers up to 12.5 months after COVID-19
Source: BMC Med. 2021 Jun 28;19:155. doi: 10.1186/s12916-021-02032-2 (PMC8237770; doi:10.1186/s12916-021-02032-2)
Supplement: Supplementary file 1 — Additional file 1: Table S1. Baseline characteristics cohort. [file 12916_2021_2032_MOESM1_ESM.docx]

**Table S1.** Baseline characteristics of the cohort of 173 primary care health care workers. During follow up, these were the sequelae (n) registered in 64 participants: anosmia (30), asthenia (18), hypogeusia (16), dyspnea (14), myalgia (7), neuralgia (6), tachycardia (6), cough (5), dry eyes (5), headache (5), blurred vision (4), nasal congestion (4), arthralgia (4), diarrhea (3), conjunctival hyperemia (3), rhinorrhea (3), chest pain (3), odynophagia (2), abdominal pain (2), tinnitus (2), rash (2), fever (1), sputum (1), hearing loss (1), dizziness (1), and alteration of consciousness (1).

| **Continuous variable** | **median (IQR)** |
| --- | --- |
| Age | 47.91 (41-58) |
|  |  |
| **Categorical variables** | **n (%)** |
| Sex |  |
| Male | 36 (20.81) |
| Female | 137 (79.19) |
| Professional category |  |
| Physician | 70 (40.46) |
| Nurse | 68 (39.31) |
| Customer and social services staff | 35 (20.23) |
| Smoking habit |  |
| Current smoker | 13 (7,51) |
| Former smoker | 31 (17,91) |
| Environmental allergies | 24 (14.12) |
| Hospitalization | 24 (13.87) |
| Fever | 131 (75.72) |
| Cough | 110 (63.58) |
| Asthenia | 133 (76.88) |
| Anorexia | 59 (34.1) |
| Diarrhea | 75 (43.35) |
| Abdominal pain | 28 (16.18) |
| Dyspnea | 64 (36.99) |
| Conjunctival hyperemia | 15 (8.67) |
| Tearing | 11 (6.36) |
| Nausea or vomiting | 22 (12.72) |
| Dry eyes | 17 (9.83) |
| Blurred vision | 9 (5.2) |
| Odynophagia | 59 (34.1) |
| Chills | 86 (49.71) |
| Sneezing | 32 (18.5) |
| Rhinorrhea | 41 (23.7) |
| Nasal obstruction | 49 (28.32) |
| Epistaxis | 10 (5.78) |
| Tinnitus | 8 (4.62) |
| Hearing loss | 3 (1.73) |
| Chest pain | 34 (19.65) |
| Tachycardia | 23 (13.29) |
| Sputum production and/or Hemoptysis | 13 (7.51) |
| Headache | 124 (71.68) |
| Dizziness | 31 (17.92) |
| Impaired consciousness | 7 (4.05) |
| Ataxia | 2 (1.16) |
| Acute cerebrovascular disease | 0 (0) |
| Seizures | 0 (0) |
| Hypogeusia and/or Anosmia | 106 (61.27) |
| Neuralgia | 8 (4.62) |
| Myalgia | 103(59.54) |
| Arthralgia | 64 (36.99) |
| Rash | 12 (6.94) |
| Vesicular lesions | 5 (2.89) |
| Maculopapules | 4 (2.31) |
| Itchy skin | 14 (8.09) |
| Thrombosis | 1 (0.58) |
| **Digestive symptoms:** diarrhea, abdominal pain, nausea or vomiting, anorexia | 100 (57.8) |
| **Otorhinolaryngology symptoms:** sneezing, rhinorrhea, nasal obstruction, epistaxis, tinnitus, hearing loss | 80 (46.24) |
| **Neurological symptoms:** impaired consciousness, ataxia, acute cerebrovascular disease, seizures, neuralgia | 17 (9.83) |
| **Ophthalmology symptoms:** conjunctival hyperemia, tearing, dry eyes, blurred vision. | 36 (20.81) |
| **Skin lesions:** rash, vesicular lesions, maculopapules, itchy skin, pseudo perniosis | 26 (15.03) |
